# Increased Expression of CXCL9, CXCL10, and CXCL11 in Epstein–Barr Virus-Associated Infectious Mononucleosis and the Role of CXCL5 as a Candidate Biomarker of Disease Severity

**DOI:** 10.3390/pathogens15050487

**Published:** 2026-05-01

**Authors:** Andrea Nikčević, Leona Radmanić Matotek, Silva Šiftar, Lorna Stemberger Marić, Goran Tešović, Snjezana Zidovec-Lepej

**Affiliations:** 1 Department for Pediatric Infectious Diseases, University Hospital for Infectious Diseases, 10000 Zagreb, Croatia; akalaba@bfm.hr (A.N.); lstemberger@bfm.hr (L.S.M.); gtesovic@bfm.hr (G.T.); 2 Department of Immunological and Molecular Diagnostics, University Hospital for Infectious Diseases, 10000 Zagreb, Croatia; leona.radmanic@gmail.com; 3 Department of Infectious Diseases, University of Zagreb School of Medicine, 10000 Zagreb, Croatia

**Keywords:** Epstein–Barr virus, infectious mononucleosis, chemokines, CXCL9, CXCL10, CXCL11, CXCL5, severity of disease scale

## Abstract

Background: Epstein–Barr virus (EBV)-associated infectious mononucleosis (IM) elicits a robust cellular immune response; however, systemic chemokine profiles in pediatric IM and their diagnostic relevance remain insufficiently characterized. This study evaluated proinflammatory chemokine expression in children with acute EBV-associated IM and its relationship with disease presence and severity. Methods: In this retrospective study, 64 children with confirmed acute EBV-associated IM and 16 healthy controls were included. Clinical severity was classified using the Severity of Mononucleosis (SOM) scale. Plasma concentrations of 12 chemokines were quantified by bead-based flow cytometry. Groups were compared using nonparametric tests, and logistic regression with cross-validation identified predictors distinguishing IM from controls. Results: CXCL9, CXCL10, and CXCL11 concentrations were significantly elevated in IM patients compared with controls across all severity strata (*p* ≤ 0.011), with no differences between SOM categories. CXCL5 concentrations were lower in severe (SOM ≥ 2) than moderate disease (*p* = 0.037). Other chemokines showed no significant differences. CXCL9 and CXCL10 effectively distinguished IM (AUC = 0.86, sensitivity = 0.71, specificity = 0.96). Conclusions: IFN-γ–inducible chemokines CXCL9–11 are markedly elevated in pediatric EBV-associated IM, irrespective of clinical severity, whereas CXCL5 may be associated with disease severity. Prospective validation of these preliminary findings is strongly warranted.

## 1. Introduction

Epstein–Barr virus (EBV), also known as human herpesvirus 4 (HHV-4), is a DNA virus belonging to the genus Lymphocryptovirus of the family Orthoherpesviridae and is associated with a variety of malignant and non-malignant diseases in humans [1]. Primary infection commonly occurs in childhood and adolescence, and approximately 90–95% of adults worldwide are EBV-seropositive [2].

Transmission of EBV is mainly person-to-person, primarily through contact with salivary secretions, with oral shedding usually persisting between 6 and 18 months after acute infection [3]. The initial contact EBV makes with oropharyngeal epithelial cells is through infected saliva, and this is the site of primary replication of the virus in B-cells of the lymphoid tissue of the oropharynx [4]. The incubation period is between four and eight weeks. During this time, lytic infection of B cells leads to dissemination to the lymphoreticular system, followed by a robust cellular immune response with activation of CD4+ and CD8+ T lymphocytes [5].

Primary EBV infection is usually asymptomatic or mild in early childhood, while in adolescents, it commonly presents as infectious mononucleosis (IM). The triad of fever, pharyngitis or tonsillitis, and lymphadenopathy characterizes IM. Atypical lymphocytosis, a hallmark of IM, typically appears one to three weeks after symptom onset and reflects a CD8+ T-cell response [6].

Clinical presentation usually begins with low-grade fever, malaise, and headache, followed by pharyngitis, lymphadenopathy, and absolute lymphocytosis with atypical lymphocytes [7].

Hepatosplenomegaly and elevated liver enzymes are common [8]. In contrast, EBV infection in young children is often asymptomatic or very mild, and when symptoms occur, respiratory manifestations are more prominent [9].

Various complications, both acute and delayed, are associated with primary EBV infection. Acute complications such as airway obstruction due to tonsillar hypertrophy are more common, while splenic rupture is rare.

Acute and delayed manifestations may complicate primary EBV infection. Acute complications, most commonly upper airway obstruction due to tonsillar hypertrophy, are relatively frequent [10], whereas splenic rupture is rare [11]. Neurological complications—including encephalitis, meningitis, acute disseminated encephalomyelitis, acute cerebellar ataxia, facial nerve palsy, Alice in Wonderland syndrome, and Guillain–Barré syndrome—occur in approximately 1–5% of patients. Cardiac involvement, such as myocarditis and pericarditis, has also been reported [12].

EBV is the first oncogenic virus described in humans. Similarly to other members of the *Orthoherpesviridae* family, EBV establishes a latent infection in target cells (B-cells, T-cells., epithelial cells and myocytes) and is considered to be an etiological co-factor for development of several human cancers: lymphoproliferative disorders (for example Burkitt’s and Hodgkin’s lymphoma) as well as non-lymphoid malignancies (nasopharyngeal carcinoma, gastric cancers, rare T- and NK- cells lymphomas and leiomyosarcoma) [13,14,15]. EBV plays an important role in the pathogenesis of lymphoproliferative disorders, such as hemophagocytic lymphohistiocytosis (HLH), X-linked lymphoproliferative disease (XLP), and post-transplant lymphoproliferative disease (PTLD). HLH is a rare but life-threatening condition, characterized by dysregulated immune system activation [16]. In immunocompromised patients, EBV can cause lymphomatoid granulomatosis or X-linked lymphoproliferative (XLP) disease in patients with selective immunodeficiency to EBV [17]. EBV is also associated with most cases of post-transplant lymphoproliferative disease (PTLD), which range from benign polyclonal B cell proliferation to malignant B cell lymphoma [18]. EBV has also been implicated in the pathogenesis of several autoimmune diseases, such as multiple sclerosis (MS) and chronic fatigue syndrome (CFS) [19,20]. Still, the relationship between EBV and CFS remains unclear [21].

Chemokines are a large family of chemotactic cytokines secreted by leukocytes, NK cells, epithelial, and endothelial cells. They act as signaling proteins and play important roles in immune cell recruitment to sites of infection or inflammation, immune system development and homeostasis, and tissue repair and wound healing. Several chemokines are implicated in the progression of malignant and autoimmune diseases. Chemokines are classified based on their primary amino-acid sequence and the arrangement of specific cysteine residues within the mature protein. Variations in the configuration of the two cysteines closest to the N terminus allow differentiation of chemokines into four subfamilies: CC, CXC, CX3C, and XC. The most studied function of the chemokine network is leukocyte migration [22]. Still, CC chemokines stimulate mainly monocytes, but also basophils, eosinophils, T-lymphocytes, and natural killer (NK) cells. The other family of CXC, known as alpha-chemokines, mainly stimulates neutrophil chemotaxis. CX3C (d-chemokines) have three amino acids between the two cysteines, and only one member of this family has been discovered to date: fractalkine (CX3CL1), which is mainly expressed on neural and microglial cells and plays a role in the protective plasticity process of synaptic scaling [23]. The fourth subfamily, called XC (or γ chemokines), is different than other chemokines in that it has only two cysteines: one N-terminal cysteine and one cysteine downstream; only two chemokines have been XCL1 (lymphotactin-α) and XCL2 (lymphotactin-β). Their main function is in the pathogenesis of rheumatoid arthritis [24].

Epstein–Barr virus manipulates chemokine production and receptor expression to establish infection and evade the immune system, regulating B cell migration and possibly contributing to the development of the above-mentioned diseases (MS, EBV-associated malignancies). This is the basis of its oncogenic potential and potential therapeutic target. Numerous studies have described chemokines induced in EBV-related conditions and diseases, primarily by LMP-1, which activates signaling pathways such as NF-κB and JNK. Nakayama T et al. LMP-1 stimulates Th-2-attracting chemokines CCL17 and CCL22 and recruits Th-1 cells through CCR5 by increasing CCL3, CCL4, and CCL5 [25]. Tsai SC et al. have shown that in EBV-immortalized lymphoblastoid cell lines, CCL3 and CCL4 were expressed at high levels [26]. Jorapur et al. have shown EBV-positive tumor cells to express high levels of chemokines CCL17 and CCL22 in vivo and in vitro, using samples from patients with lymphoblastic (Hodgkin lymphoma) and epithelial tumors (nasopharyngeal carcinoma) [27]. Ehlin-Henrikson demonstrated in vitro that EBV infection reduces CXCR4 expression on primary tonsil B cells 43 h post-infection, thereby altering chemotactic response to stromal cell-derived factor (SDF-1 alpha) [28]. However, to our knowledge, analysis of chemokine expression in EBV-associated IM in pediatric patients and its evaluation as a possible biomarker of disease have not been previously performed.

The aim of this study was to compare the expression patterns of proinflammatory chemokines in EBV-associated IM with those in healthy controls and to evaluate their potential as biomarkers for diagnosis and disease severity by analyzing their expression across different patient categories, stratified by the IM score. The panel of selected chemokines includes chemokines relevant for monocyte/macrophage responses (CCL2, CCL3, and CCL4), lymphocyte chemoattractants (CCL17 and CCL20), neutrophil recruitment mediators (CXCL1, CXCL5, and CXCL8), interferon-inducible chemoattractants for leukocytes (CXCL9, CXCL10, and CXCL11), and CCL11, which mediates the activity of eosinophils.

## 2. Materials and Methods

### 2.1. Study Design, Patients, and Data Collection

This retrospective study included 64 pediatric patients (median 13.75 years, range 4–216 months) treated for EBV-associated IM at the University Hospital for Infectious Diseases “Dr. Fran Mihaljevic”, Zagreb, Croatia (UHID), between January 2021 and December 2023, as well as 16 controls. The control group consisted of healthy children who attended a follow-up clinical visit after previous treatment for unrelated conditions (such as urinary tract infections, Kawasaki disease, or pneumonia). These children were enrolled as controls. All control participants were clinically healthy and free of acute illness at the time of study inclusion and serum sampling. Controls were frequency-matched to cases on age and sex distributions. The clinical and routine laboratory data were extracted from UHID medical records. The patients with EBV-associated IM were referred to the UHID due to the need for expanded diagnostic work-up, prolonged fever, sore throat with difficulty swallowing, general malaise, and inability to maintain adequate enteral hydration. Acute EBV-positive IM with serology (positive EBV VCA IgM or EA antibodies), and/or viremia (EBV DNA quantification in the peripheral blood) were confirmed in all patients. We excluded patients with underlying diseases and those with pre-existing EBV antibodies suggestive of past infection (i.e., EBNA IgG). Routine laboratory parameters analyzed in the study included white blood cell count, atypical lymphocytosis, bilirubin, aminotransferases, LDH, red blood cell count, hemoglobin, platelet count, and C-reactive protein. Data on clinical symptoms, presence of rash, concomitant infections, and use of antibiotics were also analyzed and used to classify patients into clinical categories based on the Severity of Mononucleosis Scale (SOM) developed by Katz et al. (2019) [29]. Informed consent was signed by the parents of all patients included in the study. The study was approved by the Ethics committee of the University Hospital for Infectious Diseases, Zagreb, Croatia, on 28 August 2019 (no. 01-1247-3-2019).

### 2.2. Classification of Patients Based on Disease Severity

Classification of patients into clinical categories based on the SOM scale is calculated based on the following criteria: clinical symptoms (sore throat that makes it difficult to swallow liquids; severe headache requiring neuroradiological examination and/or lumbar puncture; fever above 40 °C for more than 2 weeks, fever above 38 °C for more than 5 weeks; inability to leave the house during the most severe symptoms; reduced walking distance during the most severe symptoms; difficulty breathing; gastrointestinal symptoms, i.e., nausea, vomiting, loss of appetite; does not include diarrhea only); clinical signs (jaundice, pronounced painful lymphadenopathy of the neck, painful hepatosplenomegaly, petechiae of the palate) and complications (cardiac, e.g., myocarditis; hematological—thrombocytopenia < 150/mm^3^, neutropenia < 1000/mm^3^, lymphopenia < 2000/mm^3^, hemolytic anemia, hemophagocytic lymphohistiocytosis; neurological, pulmonary)—each symptom, sign, or complication carries one point; and a total score ≥ 2 indicates severe MI and a higher risk of hospitalization.

### 2.3. Chemokine Quantification

Quantification of chemokines was performed by using plasma samples stored at the biobank of the Department of Immunological and Molecular Diagnostics, UHID, at −80 °C. Quantification of 12 chemokines including MCP-1 (CCL2), MIP-1α (CCL3), MIP-1β (CCL4), eotaxin (CCL11), TARC (CCL17), MIP-3α (CCL20), GROα (CXCL1), ENA-78 (CXCL5), IL-8 (CXCL8), MIG (CXCL9), IP-10 (CXCL10), and I-TAC (CXCL11) was performed by bead-based flow cytometry using a standardized LEGENDplex™ Multi-Analyte Flow Assay Kit (LEGENDplex Human Proinflammatory Chemokine panel, BioLegend, San Diego, CA, USA). Flow cytometric analysis was performed by using the FACS Canto II instrument (Beckton Dickinson, Franklin Lakes, NJ, USA). Minimum detectable assay concentrations for the selected cytokines (pg/mL) were: CXCL8 3.4 ± 1.7, CXCL10 1.2 ± 0.9, CCL11 2.5 ± 4.8, CCL17 1.0 ± 0.9, CCL2 1.4 ± 1.4, CCL3 3.8 ± 2.6, CXCL9 0.5 ± 0.2, CXCL5 0.6 ± 0.6, CCL20 0.4 ± 0.4, CXCL1 0.5 ± 0.4, CXCL11 0.4 ± 0.3, and CCL3 0.3 ± 0.3.

### 2.4. Statistical Analysis

Categorical variables were expressed with counts and percentages, while numerical variables were reported as medians, ranges, or interquartile ranges. The normality of numerical distributions was assessed graphically and with the Shapiro–Wilk’s test. Chemokine levels were non-parametrically distributed and compared using the Kruskal–Wallis test. Dunn’s test was used for the post hoc analysis of pairwise comparisons between groups. The correlation between numerical variables was analyzed with Spearman’s correlation coefficient and the correlation test.

Patients and healthy controls were classified using binary logistic regression. To identify the most informative predictors, we applied the best subset selection algorithm, which selected CXCL9 and CXCL10 as the optimal combination. All chemokine concentrations were log_2_-transformed before modeling. The model was evaluated with 5-fold cross-validation. *p*-values were corrected for multiple testing using the Benjamini–Hochberg method. All statistical tests were two-tailed with the significance level set to 0.05. Data was analyzed using R version 4.5.1 (R Development Core Team, Vienna, Austria).

## 3. Results

### 3.1. Demographic and Clinical Data

Selected demographic and clinical features of the study cohort are presented in Table 1. A total of 28 patients (43.8%) were male, with a median age of 165 months (range 4–216 months). Ten patients (15.6%) were hospitalized for a median of 5 days (range 4–12), while others received clinical care at the pediatric outpatient clinic of UHID. Most patients in the EBV-associated IM cohort presented with pharyngitis (78.5%), bilateral cervical lymphadenitis (73.5%), and fever (70.3%), while 60.9% of patients also exhibited respiratory symptoms. Only 34.4% of patients in the cohort presented with hepatosplenomegaly. Interestingly, hypertrophic tonsils with potential for obstruction were observed in 5 patients (7.8%). All patients received supportive care, and corticosteroid treatment was initiated after a median of 5.5 days from admission in 9.4% of patients, while 14.1% received antibiotic treatment.

### 3.2. Assessment of Disease Severity

Based on the SOM Scale by Katz et al. (2019) [29], a total of 25 patients were classified as SOM = 0 (mild disease), moderate severity of disease (SOM = 1) was determined in 28 patients, while 11 patients had severe disease (SOM ≥ 2). None of the patients in the cohort had a lethal outcome or developed chronic EBV infection or hematological complications during clinical care. Comparison of selected hematological and biochemical parameters in patients with different SOM scores is presented in Table 2.

### 3.3. Analysis of Chemokine Levels in EBV-Associated IM According to Disease Severity

**Figure 1:**
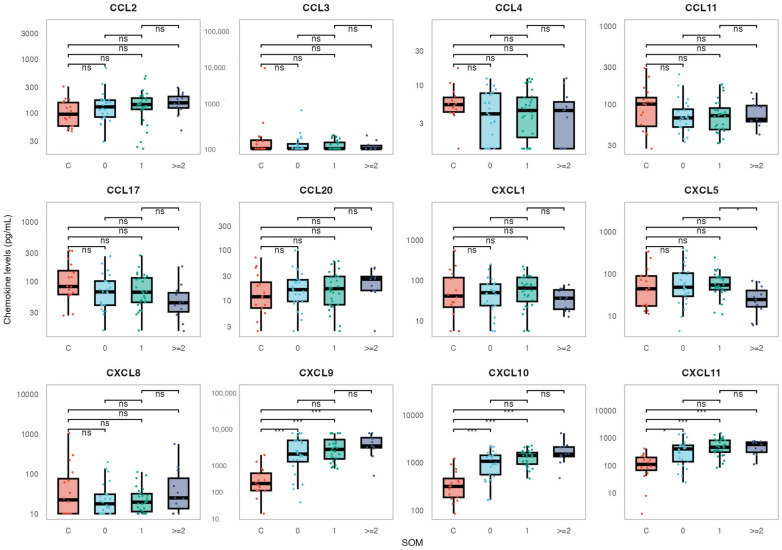
Distribution of the analyzed chemokines in IM patients, stratified by disease severity, and in controls. The boxes show the median and interquartile range of the distribution, whereas the whiskers extend to the minimum and maximum non-outlier values of the distribution. The y-axis is logarithmically scaled. ***: *p* < 0.001, *: *p* < 0.05, ns: *p* > 0.05 (Dunn’s post hoc test, *p*-values adjusted with the Benjamini–Hochberg method).

Concentrations of selected chemokines in patients with EBV-associated IM based on disease severity score SOM, as well as comparisons with healthy controls, are shown in Figure 1 (numerical values are shown in Supplemental Table S1). In patients with clinically severe disease (stage SOM2), concentrations of CXCL9 (median 3568.7 pg/mL), CXCL10 (median 1520.3 pg/mL) and CXCL11 (median 585.7 pg/mL) were significantly increased compared with controls (CXCL9 median 175.8 pg/mL, *p* < 0.001; CXCL10 median 318.3 pg/mL, *p* < 0.001.; and CXCL11 median 112.2 pg/mL; *p* = 0.002) (Figure 1, Tables S1 and Table 3). Similar patterns have been observed for patients with other disease stages. In patients with moderate disease severity (stage SOM1), concentrations of CXCL9 (median 2730.0 pg/mL), CXCL10 (median 1424 pg/mL), and CXCL11 (median 464.8 pg/mL) were also significantly increased compared with controls (*p* < 0.01 for the three chemokines). Significantly increased concentrations of CXCL9, CXCL10, and CXCL11 compared to controls have also been observed for patients with mild clinical presentation (stage SOM0; CXCL9 median 1865.6 pg/mL, *p* < 0.001; CXCL10 median 1079.5 pg/mL, *p* = 0.002; CXCL11 median 404.5 pg/mL, *p* = 0.011). Differences in CXCL9, CXCL10, and CXCL11 between patients with different disease severity scores (SOM 0, 1, and 2) were not statistically significant. Concentrations of CXCL5 were significantly elevated in patients with moderate disease (SOM1, median 54.0 pg/mL) compared to those with severe disease (SOM2, median 24.5 pg/mL, *p* = 0.037, Figure 1, Table S1). No statistically significant differences were observed in the concentrations of the chemokines CCL2, CCL3, CCL4, CCL11, CCL17, CCL20, CXCL1, and CXCL8 between IM patients and controls, nor across the different clinical severity groups (Figure 1, Tables S1 and Table 3). Correlation between concentrations of individual chemokines and between chemokine concentrations and routine laboratory parameters at admission was not statistically significant (correlation matrices shown in Figures S1 and S2).

### 3.4. Analysis of Chemokine Concentrations by Logistic Regression

The logistic regression model is shown in Table 4. For CXCL9, each doubling of the CXCL9 concentration was associated with a 1.5-fold increase in the odds of being an IM patient compared with controls (OR = 1.54, 95% CI = 1.06–2.23, *p* = 0.024). For CXCL10, each doubling of CXCL10 concentration was associated with a more pronounced five-fold increase in the odds of IM (OR = 5.15, 95% CI = 2.42–10.96, *p* < 0.001). Accuracy was 0.76, sensitivity 0.71, specificity 0.96, and AUC 0.86 (95% CI 0.81–0.93).

## 4. Discussion

The results of this study have shown increased concentrations of CXCL9, CXCL10, and CXCL11 in patients with EBV-associated IM compared with healthy controls, irrespective of clinical disease severity estimated using a clinically validated tool (SOM scale). Using logistic regression analysis, we also demonstrated the potential value of CXCL10 and CXCL9 as biomarkers for disease classification (EBV-associated IM vs. healthy controls). In addition, significantly decreased CXCL5 concentrations in IM patients with severe clinical presentation compared to patients with moderate disease have also been demonstrated. To our knowledge, few studies have described chemokine levels in acute EBV-associated IM (Fevang et al.) in pediatric patients and evaluated their value as potential disease biomarkers [30].

Literature data on EBV infection and chemokines mainly focused on in vitro and/or in vivo animal models of EBV-related hematologic (Burkitt’s lymphoma, Hodgkin’s lymphoma, nasopharyngeal cancer), or autoimmune diseases associated with EBV (multiple sclerosis, chronic fatigue syndrome), showing that chemokines CXCL8, CCL17, CCL2, and CCL20 play important roles in immunoevasion and tumorigenesis [31]. However, data on the role of chemokines and cytokines as biomarkers of diagnosis, staging, or treatment outcomes in acute EBV infection, particularly EBV-associated IM, are limited.

Chemokines CXCL9 (monokine induced by gamma interferon, MIG), CXCL10 (IFN-γ-induced protein 10, IP-10), and CXCL11 (interferon-inducible T-cell alpha chemoattractant, I-TAC) are a group of IFN-γ-inducible chemokines that share a common receptor CXCR3 and are involved in the recruitment of activated T-cells, NK-cells, NKT cells, and macrophages to sites of infection and inflammation. They also exhibit an inhibitory effect on angiogenesis and are involved in the pathogenesis of malignant and autoimmune diseases. The role of the CXCL-9, -10, and -11/CXCR3 axis in the differentiation of naive T cells into Th1 helper cells and in their migration to focal sites has been a major focus of research. The role of these chemokines in the pathogenesis of infectious and malignant diseases has also been a major focus of research. Considering that the main effector mechanisms of acute EBV infection are EBV-specific cytotoxic CD8+ T cells, which produce IFN-γ, and that IFN-γ induces chemokines CXCL9, CXCL10, and CXCL11, we hypothesized that these chemokines would be significantly elevated in patients with acute EBV IM. Our results confirm this, with strong accuracy, sensitivity, and specificity for primary EBV infection.

Fevang et al. (2021) analyzed the expression of selected cytokines and chemokines in supernatants of primary peripheral blood mononuclear cells (PBMC) cultures after in vitro mitogen stimulation as well as in the plasma of patients with acute EBV-associated IM with and without chronic fatigue at two time points (within 6 weeks since symptom onset and after 6 months of follow-up) [30]. Increased concentrations of tumor necrosis factor, interleukin-1β (IL-1β), interferon-gamma (IFN-γ), and CCL5 were observed in the supernatants of stimulated PMBCs of patients with EBV-associated IM compared to healthy controls within 6 weeks since the onset of clinical symptoms. Analysis of 27 soluble mediators of immune responses in the plasma of IM patients and controls showed significantly increased concentrations of CXCL10 and CCL-2 in IM patients within 6 weeks since disease onset compared with controls [30]. Although a direct comparison between Fevang et al. (2021) and our results is not possible due to major methodological differences (most importantly, the timing of sample collection), both studies observed increased systemic CXCL10 concentrations in EBV-associated IM [30]. Contrary to our data, Fevang et al. (2021) did not observe differences in CXCL9, CXCL11, and CXCL5 between IM patients and controls, possibly due to differences in sample collection timelines and in patients’ age (only adolescents were analyzed by Fevang et al.) [30].

Luo et al. (2025) [32] recently compared the value of IFN-γ and CXCL9 as biomarkers for diagnosis and monitoring the clinical course in EBV-associated hemophagocytic lymphohistiocytosis (HLH). IFN-γ showed superior diagnostic precision in distinguishing IM from EBV-associated HLH compared to CXCL9. However, persistently elevated CXCL9 concentrations were associated with the probability of disease reactivation. Therefore, a dual-biomarker strategy was recommended for the risk-stratified management of EBV-associated HLH, including both biomarkers for diagnostic confirmation and CXCL9 for therapeutic monitoring (Luo et al., 2025) [32]. Our study also demonstrated the value of CXCL9 as a biomarker of IM diagnosis (vs. healthy controls).

Another interesting finding was that CXCL5 was significantly elevated in patients with SOM 1 compared with SOM ≥ 2, but not in those with SOM 0 (i.e., very mild disease). The lack of a consistent gradient in CXCL5 levels across severity groups, including lower values in more severe cases, suggests that this finding may be driven by sample size limitations and temporal variability in immune responses rather than a true biological effect; thus, it should be regarded as exploratory only.

CXCL5 (epithelial neutrophil-activating peptide 78, ENA-78) binds to the CXCR2 receptor, a key mediator of neutrophil activation, and acts as a potent chemoattractant for neutrophils. However, no significant differences were observed in routine laboratory parameters between SOM groups, which may reflect that the severity scale is primarily based on clinical symptoms (e.g., high fever, lymphadenopathy, and severe pharyngitis) rather than on laboratory markers of inflammation. Further studies are warranted to clarify the role of CXCL5 in acute EBV IM, particularly given the limited sample size and the exploratory nature of the present findings. In addition, given EBV’s involvement in various hematologic and autoimmune conditions, as well as the reported association of CXCL5 with tumor progression, immunosuppressive microenvironments, and angiogenesis, future in vitro and in vivo studies may help elucidate its potential role in EBV-related malignancies.

Our study provides the first comprehensive analysis of systemic chemokine concentrations and their potential value as diagnostic markers in symptomatic primary EBV infection (e.g., IM). Several limitations of this study should be acknowledged. First, the patient cohort was recruited from a single center. It included only European participants (from Croatia), limiting the generalizability of the findings and precluding assessment of potential regional or ethnic differences. Second, the Severity of Mononucleosis (SOM) score is largely based on clinical criteria and clinician assessment, which may introduce some subjectivity. Our results should be interpreted carefully, given the risk of model overfitting, and given the small number of controls included in our study. Another important limitation is the lack of external validation in an independent patient cohort. Finally, because longitudinal follow-up was not feasible, the timing of sample collection may have influenced the results, as chemokine levels are known to be time dependent. Due to these important limitations, our results should be interpreted with caution.

In conclusion, our results suggest that CXCL9, CXCL10, and CXCL11 should be investigated further as potential predictors of primary EBV infection, and that CXCL5 may be a marker of disease severity. Further studies, preferably prospective ones with a standardized clinical, laboratory, and follow-up approach in larger cohorts, are needed to confirm our findings and possibly determine cut-off values of the abovementioned chemokines that could be relevant for their possible diagnostic application.

## Figures and Tables

**Table 1:** Demographic and clinical characteristics of the analyzed patient cohort according to disease severity.

	N (%)/Median (Range)			
Parameter	All Patients (N = 64)	SOM = 0 (N = 25)	SOM = 1 (N = 28)	SOM ≥ 2 (N = 11)
Age (months)	165 (4–216)	167 (4–200)	134 (25–212)	180 (96–216)
Gender (male)	28 (43.8%)	10 (40.0%)	12 (42.9%)	6 (54.6%)
Day of disease on admission	5 (2–21)	6.5 (2–21)	5 (2–9)	5 (2–14)
Hospitalization	10 (15.6%)	2 (8.0%)	3 (10.7%)	5 (54.6%)
Hospitalization length (days)	5 (4–12)	5 (5–5)	4 (4–4)	5 (4–12)
Fever Maximal (°C) Duration (days)	45 (70.3%) 39.0 (37.2–40.4) 5 (0–17)	15 (60.0%) 39.2 (37.2–40.4) 4 (0–17)	19 (67.9%) 39.0 (37.5–40.0) 5 (0–13)	11 (100.0%) 39.6 (38.3–40.0) 9 (4–15)
Respiratory symptoms	39 (60.9%)	15 (60.0%)	18 (63.3%)	6 (54.6%)
Angina Hyperemic pharynx Exudate on tonsils Pseudomembranous angina	51 (78.5%) 5 (7.8%) 40 (62.5%) 6 (9.4%)	18 (72.0%) 1 (4.0%) 17 (68.0%) 0 (0.0%)	25 (89.3%) 3 (10.7%) 17 (60.7%) 5 (17.9%)	8 (72.7%) 1 (9.1%) 6 (54.6%) 1 (9.1%)
Hepatomegaly	9 (14.1%)	2 (8.0%)	6 (21.4%)	1 (9.1%)
Splenomegaly	2 (3.1%)	0 (0.0%)	2 (7.1%)	0 (0.0%)
Hepatosplenomegaly	22 (34.4%)	6 (24.0%)	8 (28.6%)	8 (72.2%)
Lymphadenitis Bilateral cervical Unilateral cervical	48 (75.0%) 47 (73.5%) 1 (1.6%)	16 (64.0%) 16 (64.0%) 0 (0.0%)	22 (78.6%) 22 (78.6%) 0 (0.0%)	10 (90.1%) 9 (81.8%) 1 (9.1%)
Edema Peri-glandular edema Upper facial edema	17 (26.6%) 10 (15.6%) 7 (10.9%)	5 (20.0%) 4 (16.0%) 1 (4.0%)	7 (25.0%) 4 (14.3%) 3 (10.7%)	5 (54.6%) 2 (18.2%) 3 (27.3%)
Rash Macular Maculopapular	8 (12.5%) 1 (1.6%) 7 (10.9%)	6 (24.0%) 1 (4.0%) 5 (20.0%)	1 (3.6%) 0 (0.0%) 1 (3.6%)	1 (9.1%) 0 (0.0%) 1 (9.1%)
Hypertrophic tonsils with potential obstruction	5 (7.8%)	0 (0.0%)	3 (10.7%)	2 (18.2%)
Corticosteroid admission Day of admission Duration (days)	6 (9.4%) 5.5 (2–21) 2 (1–4)	1 (4.0%) 2 (2–2)	3 (10.7%) 6 (5–9)	2 (18.2%) 12 (3–21)
BHS coinfection	4 (6.3%)	1 (4.0%)	1 (3.6%)	2 (18.2%)
Antibiotics Phenoxymethylpenicillin First-generation cephalosporins Third-generation cephalosporins Clindamycin Doxycycline	9 (14.1%) 1 (1.6%) 2 (3.1%) 4 (6.3%) 1 (1.6%) 1 (1.6%)	4 (16.0%) 1 (4.0%) 1 (4.0%) 2 (8.0%) 0 (0.0%) 0 (0.0%)	1 (3.6%) 0 (0.0%) 1 (3.6%) 0 (0.0%) 0 (0.0%) 0 (0.0%)	4 (36.4%) 0 (0.0%) 0 (0.0%) 2 (18.2%) 1 (9.1%) 1 (9.1%)

SOM = Severity of Mononucleosis scale, BHS = β-hemolytic streptococcus group A.

**Table 2:** Routine laboratory parameters of the analyzed patient cohort.

	Median (IQR)			
Parameter	All Patients (N = 64)	SOM = 0 (N = 25)	SOM = 1 (N = 28)	SOM ≥ 2 (N = 11)
WBC count (admission) (×10^9^/L)	11.9 (8.3–14.8)	11.9 (8.1–15.6)	13.3 (9.7–16.2)	7.6 (4.9–12.1)
WBC count (maximal) (×10^9^/L)	11.9 (9.1–15.4)	11.9 (8.2–15.7)	13.4 (9.8–19.0)	11.2 (9.5–12.9)
WBC count (minimal) (×10^9^/L)	9.9 (6.7–13.5)	11.6 (7.2–14.7)	9.9 (8.0–14.1)	5.3 (4.6–9.6)
Lymphocyte count (admission) (×10^9^/L)	6.2 (4.2–8.3)	5.9 (4.0–8.6)	7.0 (5.8–9.3)	4.0 (1.5–5.1)
Lymphocyte count (maximal) (×10^9^/L)	6.8 (5.3–10.8)	6.1 (4.5–8.7)	7.3 (6.0–11.4)	6.4 (5.0–8.8)
Atypical lymphocytes (admission) (%)	22.0 (10.8–29.3)	21.5 (6.5–28.8)	25.0 (18.3–30.0)	11.5 (8–20.3)
Atypical lymphocytes (maximal) (%)	22.0 (16.0–28.5)	23.0 (13.8–28.8)	27.0 (18.5–31.0)	18.0 (14.3–32.0)
ANC (×10^9^/L)	2.5 (1.8–4.0)	2.7 (1.8–4.0)	3.2 (2.1–4.2)	1.9 (1.5–2.9)
Hemoglobin (admission) (g/L)	126 (120–140)	129 (120–137)	128 (119–142)	124 (121–139)
Hemoglobin (minimal) (g/L)	125 (117–136)	124 (119–138)	126 (117–136)	117 (107–128)
Platelet count (admission) (×10^9^/L)	178 (146–253)	248 (178–274)	172 (144–207)	133 (108–179)
Platelet count (minimal) (×10^9^/L)	176 (142–244)	236 (171–272)	170 (142–196)	123 (105–174)
CRP (admission) (mg/L)	11.2 (5.8–28.1)	10.0 (4.3–20.4)	8.5 (5.5–21.6)	26.9 (13.6–34.1)
Bilirubin (admission) (umol/L)	11 (9–19)	10 (9–12)	14 (9–12)	11 (11–20)
Bilirubin (maximal) (umol/L)	11 (9–20)	10 (9–12)	15 (11–21)	16 (11–67)
AST (admission) (U/L)	92 (43–207)	43 (39–120)	101 (48–213)	202 (124–252)
AST (maximal) (U/L)	97 (43–207)	44 (39–128)	103 (49–220)	237 (189–294)
ALT (admission) (U/L)	103 (43–258)	57 (30–166)	142 (42–271)	237 (107–371)
ALT (maximal) (U/L)	105 (43–314)	64 (34–169)	143 (48–276)	338 (253–523)
LDH (admission) (U/L)	408 (303–503)	329 (282–474)	454 (343–548)	470 (388–647)
LDH (maximal) (U/L)	409 (322–506)	335 (282–480)	454 (345–548)	514 (402–657)

IQR = interquartile range, SOM = Severity of Mononucleosis scale, WBC = white blood cells, ANC = absolute neutrophil count, CRP = C-reactive protein, AST = aspartate aminotransferase, ALT = alanine aminotransferase, LDH = lactate dehydrogenase.

**Table 3:** Pairwise comparisons of CXCL5, CXCL9, CXCL10, and CXCL11 levels between patients with increasing disease severity and controls.

	*p*			
Comparison	CXCL5	CXCL9	CXCL10	CXCL11
C vs. SOM = 0	0.646	**<0.001**	**0.002**	**0.011**
C vs. SOM = 1	0.417	**<0.001**	**<0.001**	**<0.001**
C vs. SOM ≥ 2	0.098	**<0.001**	**<0.001**	**0.002**
SOM = 0 vs. SOM = 1	0.380	0.691	0.258	0.372
SOM = 0 vs. SOM ≥ 2	0.143	0.456	0.102	0.492
SOM = 1 vs. SOM ≥ 2	**0.037**	0.691	0.326	0.988

**Table 4:** Logistic regression model classifying IM patients and controls using the analyzed chemokines. Chemokines CXCL9 and CXCL10 were chosen with the best subset selection algorithm.

Predictor	Coefficient	Std. Error	OR (95% CI)	*p*
CXCL9	0.43	0.19	1.54 (1.06–2.23)	0.024
CXCL10	1.64	0.39	5.15 (2.42–10.96)	<0.001

## Data Availability

The original contributions presented in this study are included in the article/Supplementary Materials. Further inquiries can be directed to the corresponding author.

## Supplementary Materials

The following supporting information can be downloaded at: https://www.mdpi.com/article/10.3390/pathogens15050487/s1. Table S1: Distribution of the analyzed chemokines in IM patients according to disease severity and controls; Figure S1: Correlation matrix of the analyzed chemokines in the patient cohort; Figure S2: Correlation matrix of the analyzed chemokines, routine laboratory parameters on admission, and flow cytometry parameters in the patient cohort.

## Abbreviations

The following abbreviations are used in this manuscript:

EBV	Epstein–Barr virus
IM	infectious mononucleosis
SOM	Severity of Mononucleosis Scale
BHS	β-hemolytic streptococcus group A
IQR	interquartile range
WBC	white blood cells
ANC	absolute neutrophil count
CRP	C-reactive protein
AST	aspartate aminotransferase
ALT	alanine aminotransferase
LDH	lactate dehydrogenase
