# Supplementary material for: Increased Expression of CXCL9, CXCL10, and CXCL11 in Epstein–Barr Virus-Associated Infectious Mononucleosis and the Role of CXCL5 as a Candidate Biomarker of Disease Severity
Source: Pathogens. 2026 May 1;15(5):487. doi: 10.3390/pathogens15050487 (PMC13209317; doi:10.3390/pathogens15050487)
Supplement: Supplementary file 1 [file pathogens-15-00487-s001.zip › pathogens-4232085-supplementary.pdf]

Table S1. Distribution of the analysed chemokines in IM patients according to disease severity and controls.

	Median (IQR)				
**Chemokine**	**Controls**	**SOM = 0**	**SOM = 1**	**SOM >= 2**	**p**
CCL2	95.9 (57.2-156.8)	130.5 (84.2-173.4)	143.5 (117.0-189.8)	154.5 (123.3-203.4)	0.236
CCL3	50.7 (50.7-84.6)	50.7 (50.7-67.4)	50.7 (50.7-73.5)	50.7 (50.7-61.0)	0.902
CCL4	5.4 (4.3-6.9)	4.1 (1.3-7.8)	4.5 (2.0-6.9)	4.5 (1.3-6.0)	0.541
CCL11	101.9 (52.6-122.7)	67.2 (51.3-86.7)	71.7 (47.6-90.1)	64.5 (60.2-96.6)	0.537
CCL17	82.0 (60.2-153.0)	66.6 (39.7-101.3)	65.9 (44.5-114.7)	43.8 (31.0-64.6)	0.087
CCL20	11.0 (7.1-23.4)	16.4 (9.5-25.7)	17.1 (8.2-29.9)	26.4 (15.8-29.7)	0.496
CXCL1	41.3 (22.4-118.8)	49.9 (23.9-81.3)	65.2 (30.4-118.7)	36.5 (19.5-58.5)	0.402
CXCL5	44.1 (17.2-89.1)	48.0 (29.4-104.0)	54.0 (41.9-82.6)	24.5 (16.6-41.2)	**0.046**
CXCL8	22.6 (10.2-78.6)	18.0 (10.2-31.2)	19.7 (11.5-32.2)	25.3 (14.2-86.9)	0.732
CXCL9	175.8 (99.4-405.2)	1865.6 (1026.8-5619.0)	2730.0 (1275.7-6276.8)	3568.7 (3105.3-6977.7)	**<0.001**
CXCL10	318.3 (187.6-476.5)	1079..5 (563.9-1437.1)	1424.5 (937.7-1637.0)	1520.3 (1352.3-2143.0)	**<0.001**
CXCL11	112.2 (68.3-198.2)	404.5 (140.0-545.7)	464.8 (307.8-826.6)	585.7 (309.4-708.9)	**<0.001**

IQR = interquartile range, SOM = Severity of Mononucleosis scale

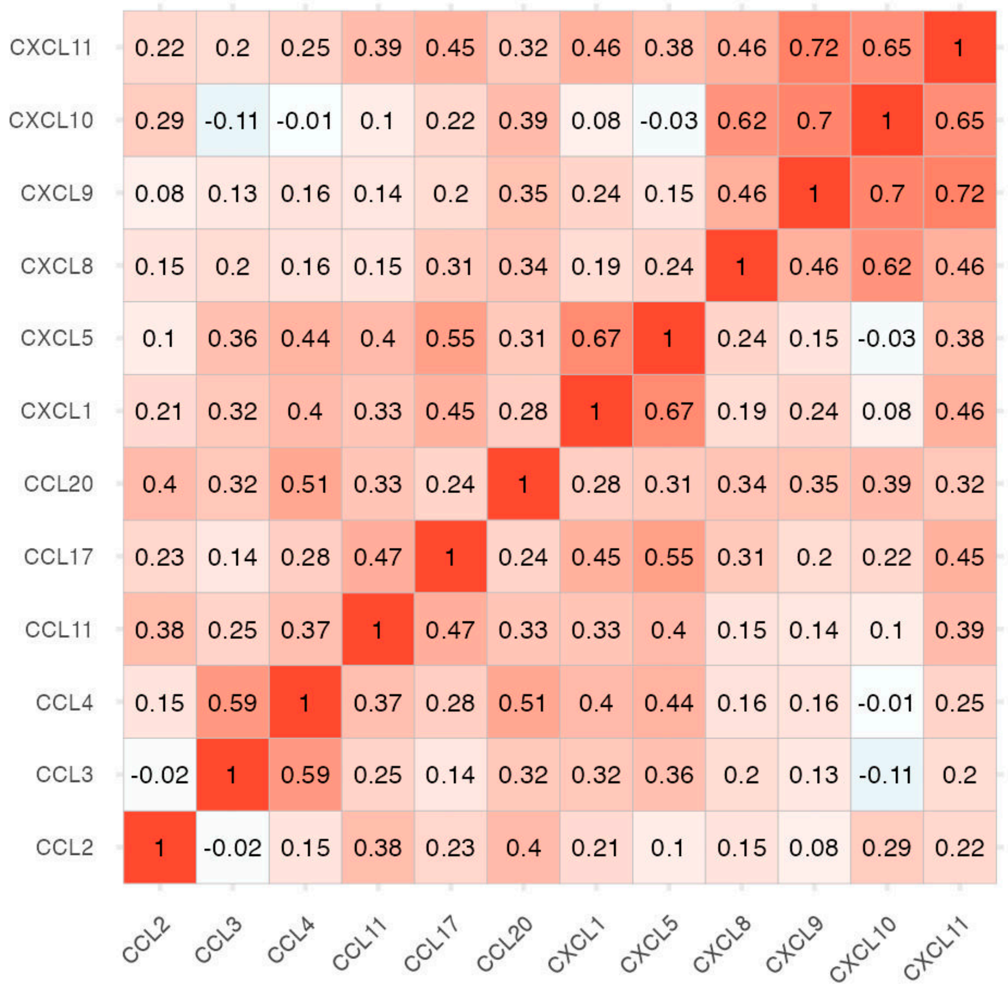

Figure S1. Corelation matrix of the analysed chemokines in the patient cohort. Presented values correspond to Spearman's correlation coefficient. Color intensity is proportional to the Spearman correlation coefficient.

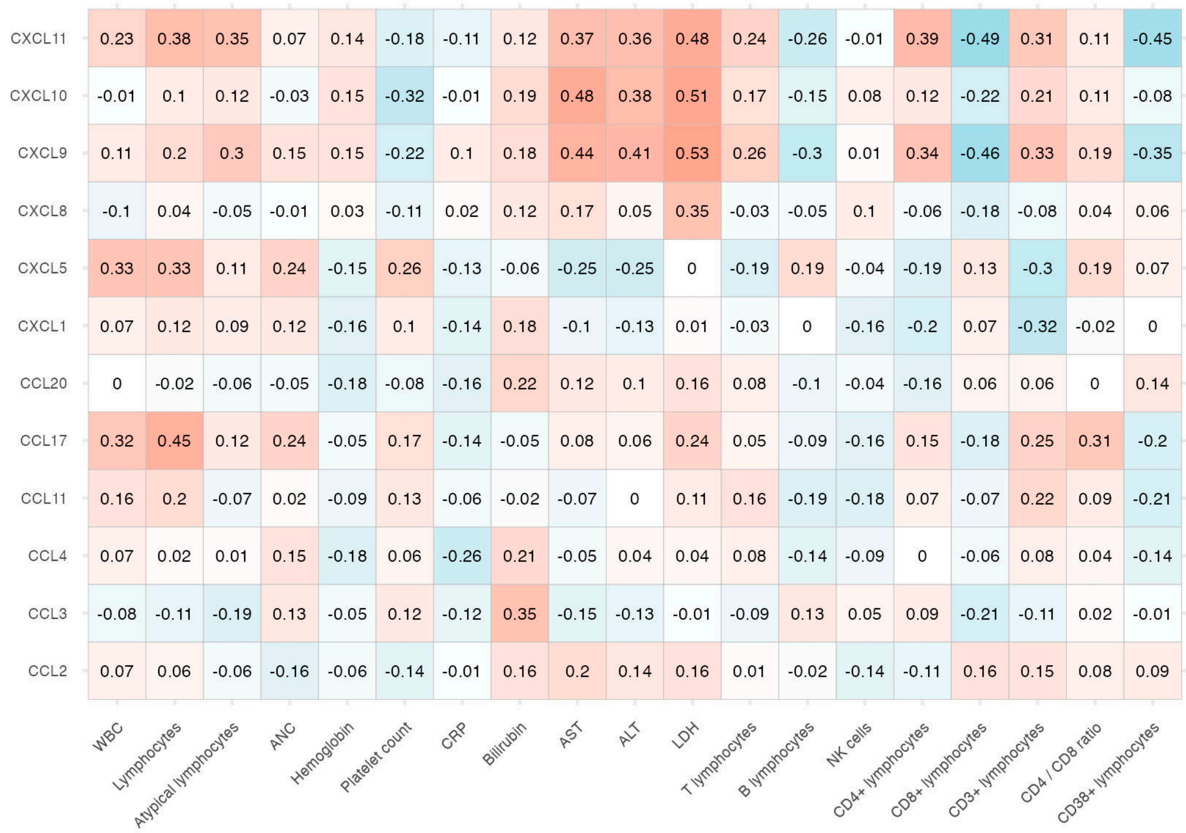

Figure S2. Corelation matrix of the analysed chemokines, routne laboratory parameters on admission and flow cytometry parameters in the patient cohort. Presented values correspond to Spearman's correlation coefficient. Color intensity is proportional to the Spearman correlation coefficient.
